# AIE-active theranostic system: selective staining and killing of cancer cells†
†Electronic supplementary information (ESI) available: Further details of TPE-IQ, characterization of TPE-IQ-2O, optimization of staining conditions and demonstration of working mechanism. See DOI: 10.1039/c6sc04947h


**DOI:** 10.1039/c6sc04947h

**Published:** 2016-12-13

**Authors:** Chen Gui, Engui Zhao, Ryan T. K. Kwok, Anakin C. S. Leung, Jacky W. Y. Lam, Meijuan Jiang, Haiqin Deng, Yuanjing Cai, Weijie Zhang, Huifang Su, Ben Zhong Tang

**Affiliations:** a HKUST Shenzhen Research Institute , No. 9 Yuexing 1st RD, South Area, Hi-tech Park Nanshan , Shenzhen 518057 , China; b Division of Biomedical Engineering , Department of Chemistry , Hong Kong Branch of Chinese National Engineering Research Center for Tissue Restoration and Reconstruction , Institute for Advanced Study , Institute of Molecular Functional Materials , State Key Laboratory of Molecular Neuroscience , The Hong Kong University of Science and Technology , Clear Water Bay , Kowloon , Hong Kong , China . Email: tangbenz@ust.hk; c Guangdong Innovative Research Team , SCUT-HKUST Joint Research Laboratory , State Key Laboratory of Luminescent Materials and Devices , South China University of Technology , Guangzhou 510640 , China

## Abstract

Cancer is the leading cause of death worldwide. With the advantages of low cost, high sensitivity and ease of accessibility, fluorescence imaging has been widely used for cancer detection in the scientific field. Aggregation-induced emission luminogens (AIEgens) are a class of synthesized fluorescent probes with high brightness and photostability in the aggregate state. Herein, a new positively-charged AIEgen, abbreviated as TPE-IQ-2O, is designed and characterized. TPE-IQ-2O not only can distinguish cancer cells from normal cells with high contrast with the aid of the difference in mitochondrial membrane potential as well as the quantity of mitochondria, but it also works as a promising photosensitizer to kill cancer cells through generation of reactive oxygen species upon white light irradiation, thus making it a promising AIE theranostic system.

## Abstract 2

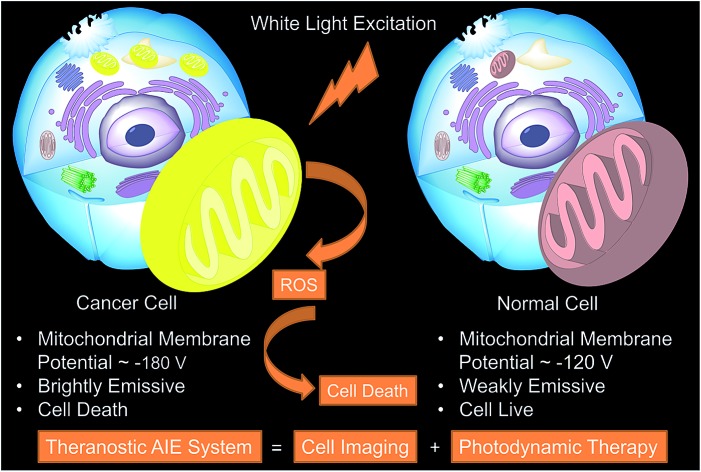
A mitochondrion-specific AIEgen is a theranostic molecule, with the function of lighting up and killing cancer cells rather than normal cells.

## Introduction

Cancer is the leading cause of morbidity and mortality worldwide. Due to the rapid growth and aging of the population, the incidence of cancer is increasing. The number of new cases is estimated to rise by about 70% over the next two decades. Every year, enormous resources have been devoted in medical research to learn the causes of cancer and develop new techniques as well as effective cancer drugs for diagnosis and treatment. In fact, early detection of cancer before its metastasis can increase the survival rate of patients. However, it is not easy to diagnose cancer as the tumor size needs to be large enough to observe. To date, diagnostic tests, including physical examination, biopsy, imaging tests and endoscopy, have been commonly used. Among them, ultrasound, X-ray, computed tomography (CT) and magnetic resonance imaging (MRI) have been widely used in clinics as imaging tools for locating the tumor cells. Unfortunately, these techniques show some drawbacks and limitations.^1^ For example, the sensitivity of ultrasound is poor because of its low signal-to-noise ratio. The frequency used in X-rays or CT scans is limited due to the health risk caused by radiation overdose. On the other hand, the instrumental and operational costs for CT and MRI are relatively expensive for hospitals and patients.

Compared to conventional imaging techniques, fluorescence imaging possesses the advantages of low cost, high sensitivity and good accessibility.^2^–^4^ It has been utilized to monitor spatiotemporal distribution of molecular processes and biological structures in a real time manner. A variety of organic fluorophores such as Nile red, BODIPY and rhodamine have been developed for cell and tissue imaging. These fluorophores are often used in solution with a low concentration because their emissions are quenched at a high concentration due to the aggregation-caused quenching (ACQ) effect. The ACQ effect greatly restricts the working concentration of conventional dyes, thus limiting their further applications in super-sensitive analysis and long-term monitoring as they are easily photo-blenched upon photoexcitation.^5^ Recently, our group proposed a new photophysical phenomenon of aggregation-induced emission (AIE) in a class of luminogens with rotor-like structures.^6^ In contrast to conventional ACQ dyes, luminogens with AIE characteristics (AIEgens) are weakly emissive when dissolved in solution, but show fluorescence in the aggregate state. This feature renders AIEgens to function at a high concentration, making them promising candidates for long-time tracking or analyte recognition with superb sensitivity and photostability.^7^–^12^

To achieve better specificity to cancer cells, fluorescent dyes or nanoparticles are usually conjugated with tumor-specific biomarkers, such as antibodies.^13^ For example, cetuximab is a monoclonal antibody that can selectively target to an epidermal growth factor receptor, which is overexpressed in colorectal and breast cancer cells.^14^,^15^ Although conjugation of biomolecules to fluorescent dyes can improve the imaging contrast between cancer and normal cells, this method involves complicated molecular design, synthetic step and product purification procedure. Because of the high specificity endowed by the biomarkers, these fluorescent probes are not able to diagnose diverse cancer cells.^16^ Thus, it would be more fascinating if a fluorescent dye with a simple structure can be developed at a low cost for differentiating normal cells and cancers cells with high imaging contrast.

Theranostics is a concept proposed in 2002, which combines diagnostic imaging and therapy.^17^ Since then, many materials showing the functionality of photodynamic therapy (PDT), chemotherapy or radiation therapy are incorporated into different imaging agents.^18^,^19^ However, chemotherapy and radiation therapy can bring severe side effects. On the other hand, PDT is a clinically approved therapeutic technique for eliminating malignant tumor cells with minimum invasion.^20^–^22^ The procedure involves a photosensitizer selectively located in the tumor sites. Upon light irradiation, the photosensitizer generates reactive oxygen species (ROS), which induces cell apoptosis or damages the microvasculature. However, existing photosensitizers suffer from the problems of long irradiation time, poor photostability and therapeutic efficiency.

**Fig. 1:**
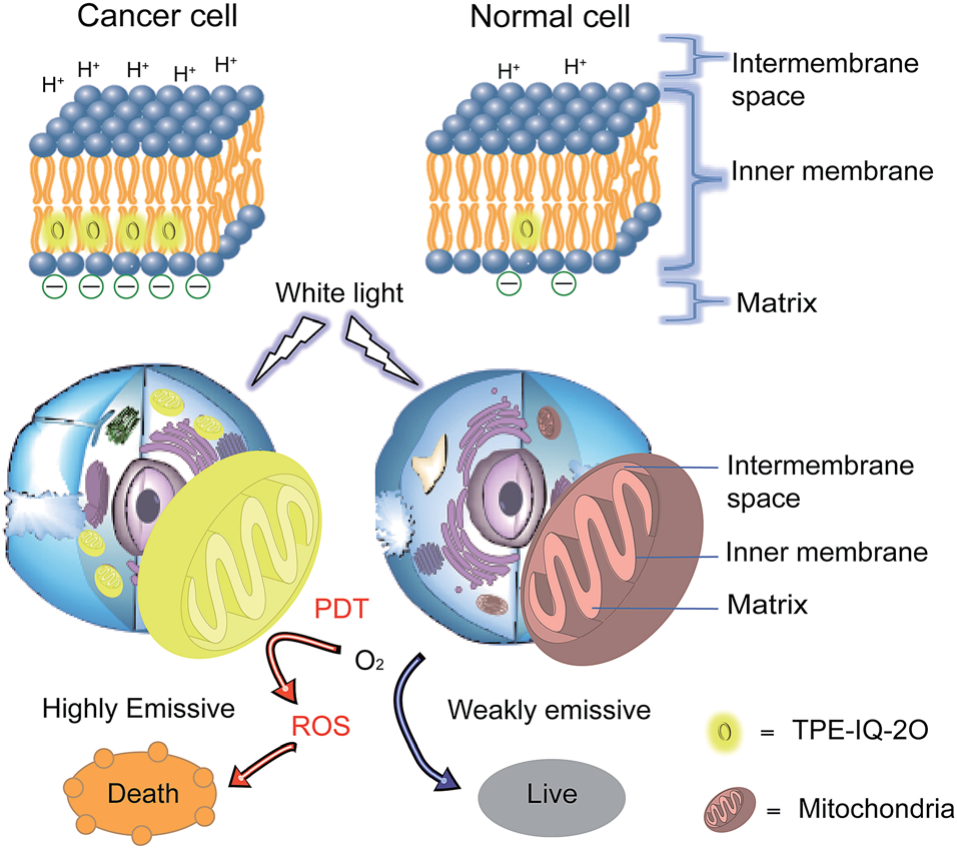
Schematic illustration of TPE-IQ-2O as a fluorescent bioprobe for selective mitochondrion imaging in cancer cells and a photosensitizer for photodynamic therapy (PDT). ROS: reactive oxygen species.

Recently, a mitochondrion-targeting isoquinolinium-based AIEgen with a PDT effect, called TPE-IQ, was reported by us (see ESI, Fig. S1†).^23^ TPE-IQ can illuminate mitochondria in live cells with superb selectivity and generate ROS to induce cell apoptosis. TPE-IQ is thus a promising theranostic system. However, its absorption is located at the ultraviolet region, which may cause undesirable effects to normal tissues upon long time exposure. Motivated by the previous success, we intend to develop a theranostic system that can selectively stain and kill cancer cells by using white light irradiation. Herein, we modified the molecular structure of TPE-IQ by introducing electron-donating methoxy group. This positively-charged AIEgen with yellow fluorescence, denoted as TPE-IQ-2O, not only can distinguish cancer cells from normal cells based on the difference in the mitochondrial membrane potential and quantity of mitochondria, but also work as a potential photosensitizer to induce cancer cell death through ROS generation upon white light irradiation, demonstrating creating a new promising theranostic AIE system (Fig. 1).

## Results and discussion

### Optical properties of TPE-IQ-2O

**Fig. 2:**
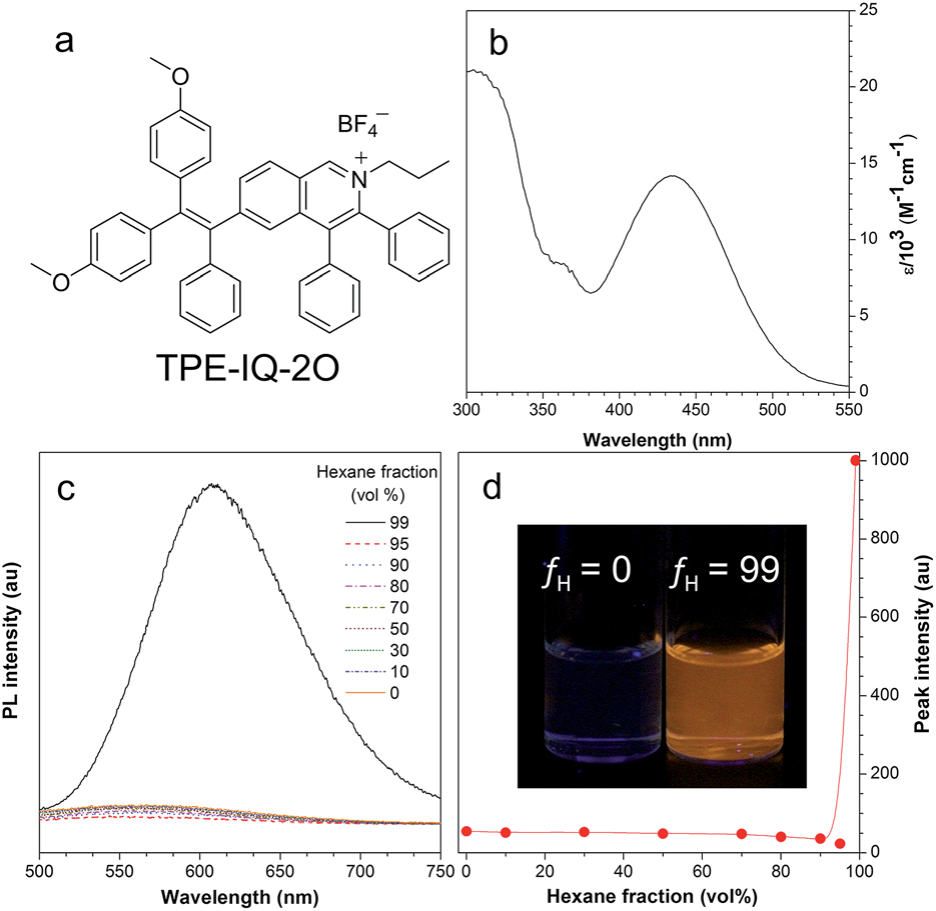
(a) Molecular structure of TPE-IQ-2O. (b) UV-vis spectrum of TPE-IQ-2O in DMSO. (c) Photoluminescence (PL) spectra of TPE-IQ-2O in THF/hexane mixtures with different hexane fractions (*f*_H_). (d) Plot of relative PL intensity (*I*/*I*_0_) *versus f*_H_. Inset: fluorescent photos of THF/hexane mixtures of TPE-IQ-2O at *f*_H_ = 0 and 99 vol% taken under 365 nm UV illumination from a hand-held UV lamp. Concentration: 10 μM; *λ*_ex_: 430 nm.

The synthetic route to TPE-IQ-2O was depicted in Fig. S2 in the ESI.† The structure of TPE-IQ-2O was characterized by NMR and mass spectroscopies with satisfactory results (see ESI, Fig. S3–S5†). The photo-physical properties of TPE-IQ-2O were then studied. The UV-vis absorption spectrum of TPE-IQ-2O was recorded in DMSO solution and is shown in Fig. 2b. TPE-IQ-2O exhibits a strong absorption band peaked at 430 nm (*ε*: ∼15 000 M^–1^ cm^–1^), which is 30 nm red-shifted from that of TPE-IQ (*λ*_abs_: 400 nm). The absorption red-shift is attributed to introducing electron-rich methoxy groups to the TPE core. TPE-IQ-2O inherits the AIE feature from TPE-IQ, as evidenced by the photoluminescence (PL) spectra measured in the solution and aggregate states (Fig. 2c and d). TPE-IQ-2O emits faintly in pure THF solution. Its emission remains low even when a large amount of hexane (95 vol%) is added to the THF solution. Upon further increasing the hexane fraction to 99 vol%, a strong emission peak at 620 nm was detected and bright yellow fluorescence was observed under 365 nm UV illumination from a hand-held UV lamp (Fig. 2d). Such a distinct emission difference is possibly due to formation of aggregates of TPE-IQ-2O as its solubility in hexane is poor. The quantum yield (QY) in pure THF and in the THF/hexane mixture (1 : 99, v/v) was 0.6% and 13.1%. Clearly, the PL and QY results demonstrated that TPE-IQ-2O exhibits the AIE property.

### TPE-IQ-2O can specifically target the mitochondria

**Fig. 3:**
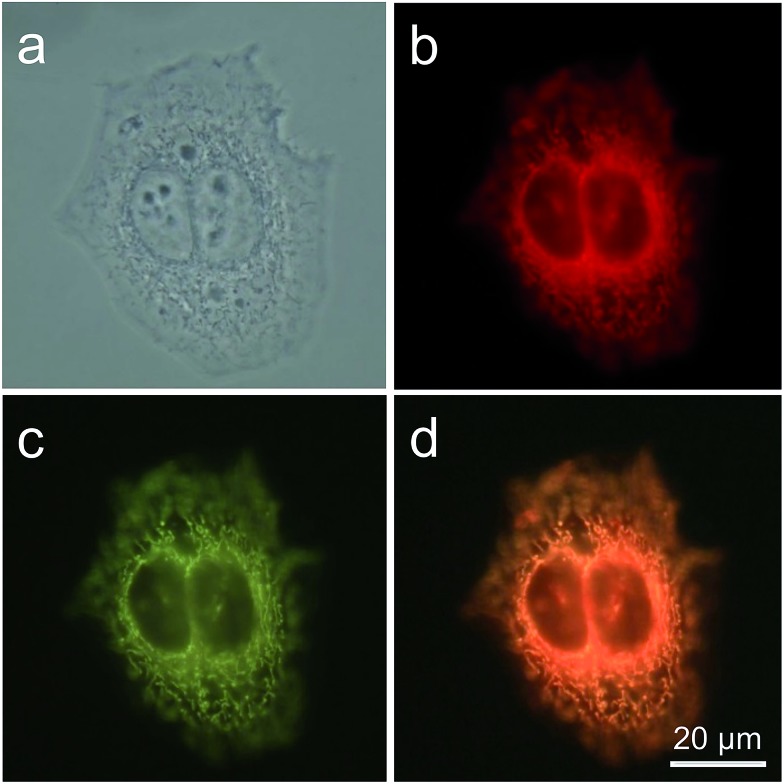
Co-localization imaging of HeLa cells stained with MitoTracker Red and TPE-IQ-2O. (a) Bright-field image and (b and c) fluorescent images of HeLa cells stained with (b) MTR (50 nM) and (c) TPE-IQ-2O (200 nM) for 20 min. (d) Merged image of panel (b) and (c). *λ*_ex_: 540–580 nm (MTR) and 400–440 nm (TPE-IQ-2O); scale bar = 20 μm.

TPE-IQ is shown to be a mitochondrion-targeting AIEgen. To verify whether this also holds true for TPE-IQ-2O, HeLa cells were used as a cell model and were co-stained with TPE-IQ-2O and MitoTracker Red (MTR, 50 nM) for 20 min. At different excitation wavelengths, red fluorescence from MTR and greenish yellow fluorescence from TPE-IQ-2O are readily observed (Fig. 3b and c). The correlation coefficient for the two images are calculated to be 98% (Fig. 3d), suggesting that TPE-IQ-2O can specifically target the mitochondria. On the contrary, when the HeLa cells were co-stained with TPE-IQ-2O and other commercial organelle-targeting dyes such as DAPI and LysoTracker Red (see ESI, Fig. S6†). Neither photo with blue emission or red emission overlapped well with the greenish yellow emission of TPE-IQ-2O, suggesting that TPE-IQ-2O localizes specifically in the mitochondria but not in other organelles.

### TPE-IQ-2O differentiates cancer cells from normal cells

**Fig. 4:**
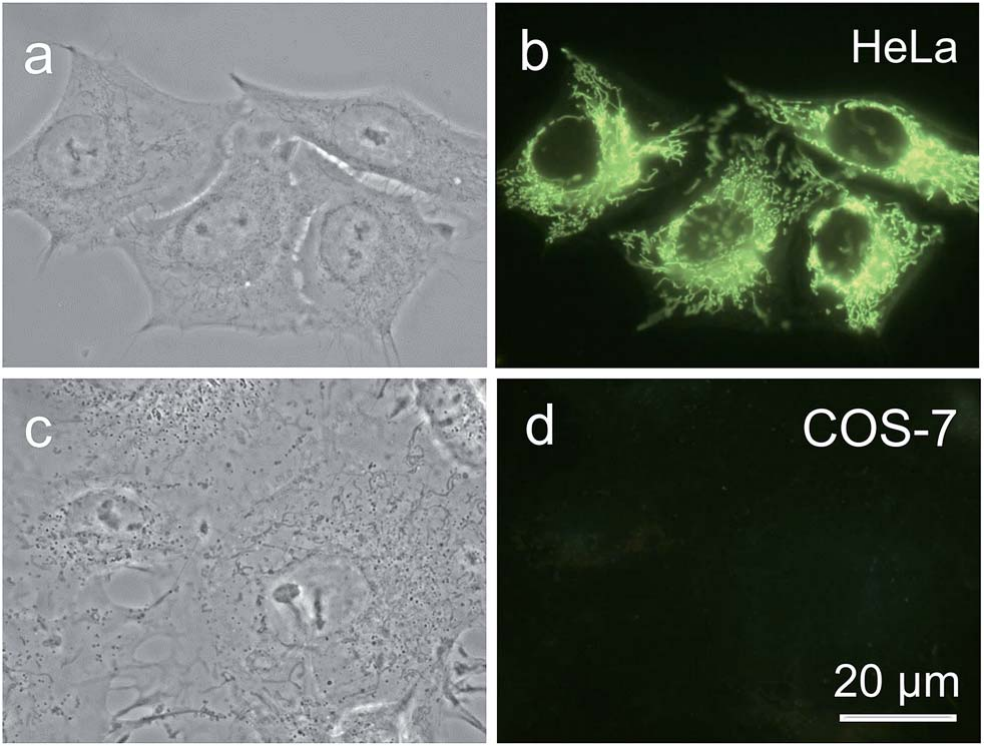
Differentiation of cancerous HeLa cells from normal COS-7 cells by TPE-IQ-2O. (a and c) Bright-field and (b and d) fluorescent images of (a and b) HeLa cells and (c and d) COS-7 cells incubated with 200 nM of TPE-IQ-2O for 20 min.

It is reported that cancer cells shows a higher mitochondrial membrane potential (MMP) than normal cells with a difference of at least 60 mV for their more active metabolism.^24^ In addition, cancer cells hunger for food in order to supply enough energy for metabolism. Thus, they strive for more substances in the surroundings. Due to the stronger electrostatic interaction, we speculate that TPE-IQ-2O will be internalized and accumulated more in cancer cells than normal cells, giving rise to stronger fluorescence. To examine this hypothesis, cancerous HeLa cells and normal COS-7 cells were incubated with TPE-IQ-2O under the same conditions followed by observation under fluorescence microscopy. As shown by the fluorescent images in Fig. 4, clear and fine mitochondrion structures with strong greenish yellow fluorescence was observed in HeLa cells. In sharp contrast, almost no fluorescence was observed in COS-7 cells. This result is very promising and it motivates us to carry out more experiments to obtain more detailed information on such differentiation.

**Fig. 5:**
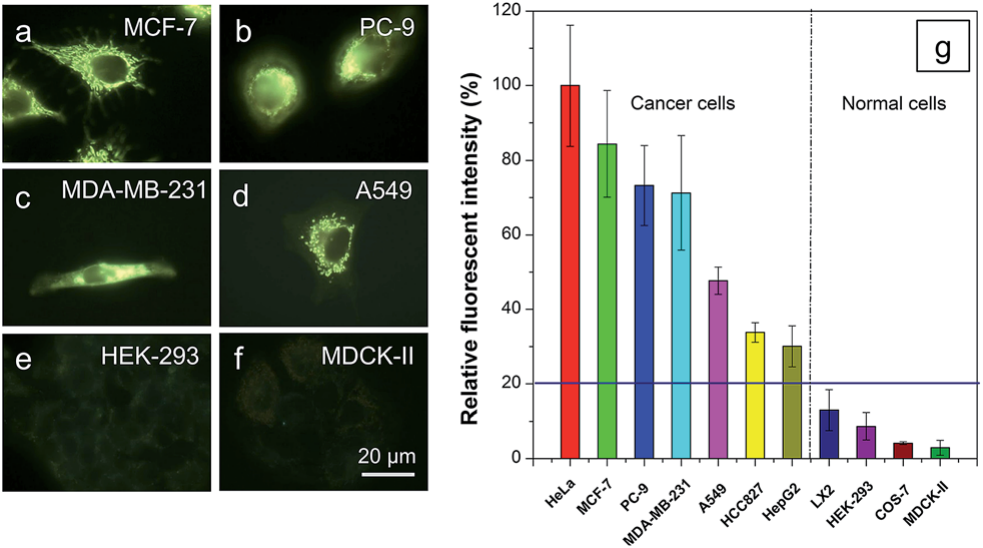
Differentiation of cancer cells from normal cells by TPE-IQ-2O. (a–f) Fluorescent images of different (a–d) cancer cells and (e–f) normal cells stained with TPE-IQ-2O (200 nM) for 20 min. (g) Relative fluorescent intensity of different cells incubated with TPE-IQ-2O (200 nM) for 20 min. *λ*_ex_: 400–440 nm.

We first optimized the staining conditions to achieve maximum emission contrast between the two cell types. Through a series experiments, we finally found that the best result was obtained at a dye concentration of 200 nM and a staining time of 20 min (see ESI, Fig. S7–S12†). Second, to verify that TPE-IQ-2O could be used as a selective fluorescent probe for all cancer cells, different cell lines were employed and stained with TPE-IQ-2O. All the cancerous cells (MDA-MB-231, MCF-7, PC-9, A549, HCC-827 and HepG2) stained by TPE-IQ-2O exhibit greenish yellow emissions (Fig. 5a–d and S13 of the ESI†).

Comparatively, normal cells, including LX-2, HEK-293 and MDCK-II, display a much weaker fluorescence (Fig. 5e and f and S13 of the ESI†). The PL intensity from all dye-stained cells was further processed and compared using MATLAB software, and the results are shown in Fig. 5g. Obviously, cancer cells fluoresce much stronger than normal cells, demonstrating that TPE-IQ-2O can preferentially target and light up a wide range of cancer cells. This makes it a potentially effective fluorescent probe for specific staining of cancer cells.

**Fig. 6:**
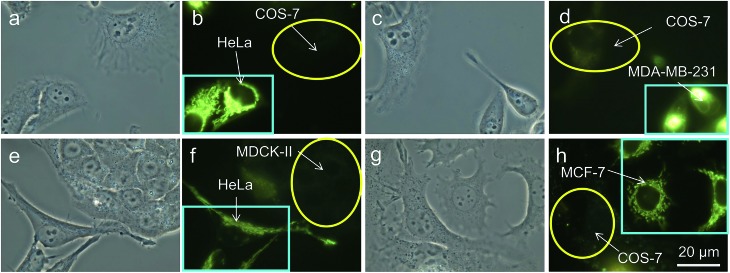
Co-culture of different combinations of cancer cells and normal cells in culture medium with TPE-IQ-2O. (a, c, e and g) Bright-field images and (b, d, f and h) corresponding fluorescent images of (a and b) HeLa and COS-7 cells, (c and d) MDA-MB-231 and COS-7 cells, (e and f) HeLa and MDCK-II cells and (g and h) MCF-7 and COS-7 cells incubated in Dulbecco's Modified Eagle Medium (DMEM) with TPE-IQ-2O. Light blue rectangles represent cancer cells and yellow circles represent normal cells. All the images share the same scale bar: 20 μm.

Thirdly, in an effort to mimic the physiological environment in individual tissues, cancer cells (HeLa cells or MDA-MB-231 cells) and normal cells (COS-7 cells or MDCK-II cells) were co-cultured in the same dish.^25^ After 24 h, both cancer and normal cells in the same dish were stained with 200 nM of TPE-IQ-2O for 20 min. Interestingly, only cancer cells show bright emission while their normal cousins emit no light at all (Fig. 6). Shortening the staining time to 10 min exerts no effect on the recognition ability of TPE-IQ-2O (Fig. 6e–h), illustrating that these molecules can selectively accumulate only in the mitochondria of cancer cells even when both cell types are co-cultured together.

Lastly, in the early-stage of the disease, a small amount of cancer cells are usually surrounded by a large number of normal cells. Thus, to mimic the actual environment, cancer cells (HeLa cells) and normal cells (COS-7 cells) with ratios of 1 : 1, 1 : 5, 1 : 50 and 1 : 100 were co-cultured in Petri-dishes and then incubated with TPE-IQ-2O. Result shows that even when the population ratio between the two cells changes from 1 : 1 to 1 : 100, we can still observe tiny greenish-yellow emission spots from dye-stained cancer cells (see ESI, Fig. S14†). Collectively, all the results support that TPE-IQ-2O is a promising candidate for searching for rare cancer cells in normal tissues.

### Does TPE-IQ-2O accumulate in cancer cells based on elevated MMP cells over normal cells?

**Fig. 7:**
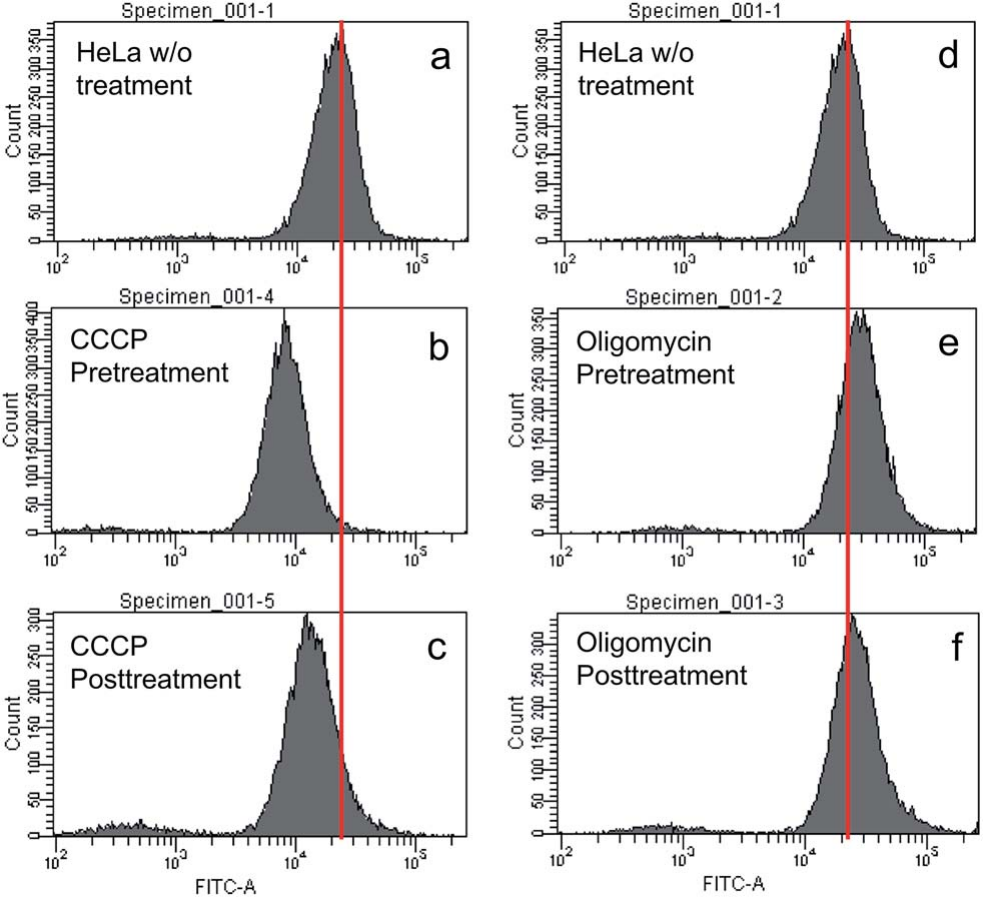
Flow cytometry analysis of HeLa cells incubated with TPE-IQ-2O in the (a and d) absence and (b, c, e and f) presence of (b) CCCP pre-treatment, (c) CCCP post-treatment, (e) oligomycin pre-treatment and (f) oligomycin post-treatment. Concentration: TPE-IQ-2O (5 μM), CCCP (20 mM) and oligomycin (10 mg mL^–1^); treatment (incubation) time: 30 min; *λ*_ex_: 405 nm.

To understand the working principle of TPE-IQ-2O, carbonyl cyanide *m*-chlorophenyl hydrazone (CCCP) and oligomycin were employed to alter the MMP of HeLa cells.^26^ The treated or untreated cells were then subjected to flow cytometry measurement, which is a powerful technique for quantitative analysis of a large number of samples and provides comprehensive information on the overall distribution of cells through analysis of physical characteristics, such as relative size and fluorescence intensity. When the HeLa cells were first pretreated with CCCP (a commonly used chemical for decreasing MMP) followed by staining with TPE-IQ-2O, weaker fluorescence was the result (Fig. 7a and b). This should be attributed to the decrease in the electrostatic attraction between TPE-IQ-2O and HeLa cells upon CCCP treatment. The HeLa cells also exhibit weaker fluorescence when they are treated with CCCP after staining (Fig. 7a and c), but the number is less than that in Fig. 7b. The TPE-IQ-2O molecules may leak out from the cells when the MMP becomes lower. On the contrary, when the HeLa cells were pretreated with oligomycin, which is an agent for rising MMP, stronger fluorescence was observed (Fig. 7d and e). No obvious emission change was detected when the dye-labelled HeLa cells were further treated with oligomycin (Fig. 7d and f). It is possibly due to the fact that no more free TPE-IQ-2O molecules are present in the culture medium for further staining. The quantitative fluorescence intensity of HeLa cells with diverse MMP is shown in Fig. S15 of the ESI.† In addition, dead HeLa cells with no MMP emit a much weaker fluorescence than the live ones (see ESI, Fig. S16†), indicating that MMP is essential for the specific staining of TPE-IQ-2O. It now becomes clear that why the cancer cells show a stronger fluorescence than normal cells.

To validate whether the fluorescence of different cells stained with TPE-IQ-2O is correlated with their MMP, a MMP-dependent molecule, JC-1, was employed for such investigation.^27^,^28^ While JC-1 emits green fluorescence in its monomer form at low MMP, it aggregates at high MMP and fluoresces in the red light region (see ESI, Fig. S17a–c†). Based on this property, the intensity ratio between red and green fluorescence of JC-1 can reflect the MMP of cells. HeLa, A549 and COS-7 cells were stained with JC-1 and the fluorescence in the red and green channels were collected by flow cytometry. By calculating the red/green ratio, the MMP of the cells is ranked. According to Table S2 of ESI,† while HeLa cells show the highest MMP, COS-7 cells possess the lowest value. Such a result is in good agreement with the emission intensity observed in the fluorescent images (see the ESI, Fig. S18†).

**Fig. 8:**
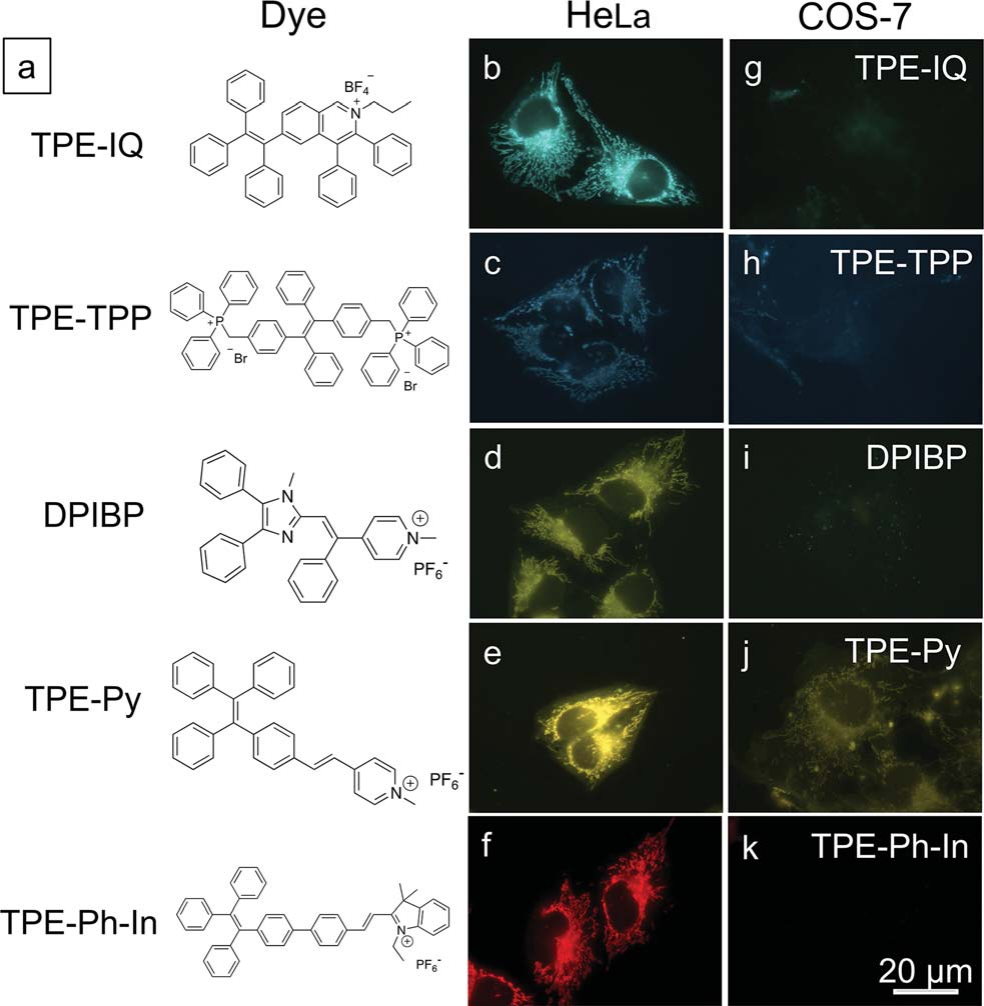
Differentiation of cancerous HeLa cells from normal COS-7 cells by various mitochondrion-specific AIEgens. (a) Chemical structures of different mitochondrion-targeting AIEgens. (b–k) Fluorescent images of (b–f) HeLa cells and (g–k) COS-7 cells stained with (b and g) TPE-IQ (200 nM, 20 min), (c and h) TPE-TPP (5 μM, 15 min), (d and i) DPIBP (5 μM, 30 min), (e and j) TPE-Py (5 μM, 15 min) and (f and k) TPE-Ph-In (5 μM, 30 min). Scale bar = 20 μm.

Based on the above findings, MMP is an important parameter for TPE-IQ-2O to differentiate cancer cells from normal cells. We speculate that the mitochondrion-specific probes based on positively-charged AIEgens preferentially target cancer cells over normal cells. To prove this speculation, five AIE-based mitochondrial probes with different chemical structures and emission colours were investigated (Fig. 8a).^29^–^31^ From the fluorescent images shown in Fig. 8b–k, all the probes preferentially light up the cancerous HeLa cells. In sharp contrast, only weak fluorescence was observed in normal COS-7 cells.

### TPE-IQ-2O is a promising photosensitizer for photodynamic therapy

**Fig. 9:**
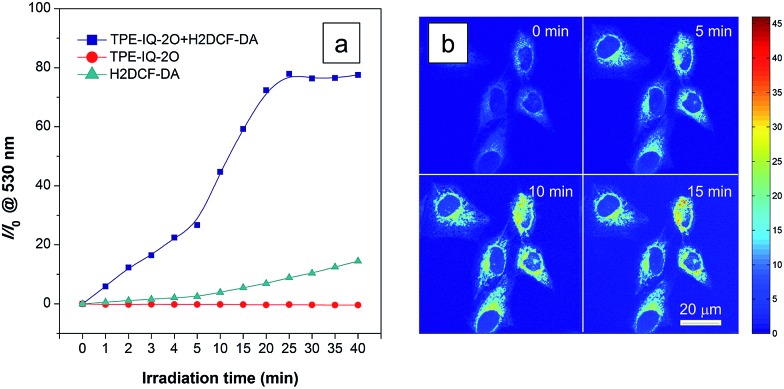
ROS generation upon white light irradiation. (a) Relative fluorescence intensity (*I*/*I*_0_) of TPE-IQ-2O, H_2_DCF-DA, and their mixture in the presence of white light irradiation for different times. *I*_0_ is the fluorescence intensity before light irradiation. Concentration: 10 μM (TPE-IQ-2O) and 5 μM (H_2_DCF-DA); *λ*_ex_: 488 nm. (b) Confocal images of HeLa cells co-stained with TPE-IQ-2O (1 μM) and H_2_DCF-DA (1 μM) at different time under continuous white light illumination.

Apart from being a mitochondrial probe, TPE-IQ-2O can also work as a photosensitizer, generating reactive oxygen species (ROS) upon light irradiation for PDT. Since TPE-IQ-2O absorbed strongly in the visible light region, white light was used as the excitation light source. H_2_DCF-DA was used as a ROS indicator, which emits fluorescence at around 530 nm in the presence of ROS.^32^ In the presence of TPE-IQ-2O, the fluorescence of H_2_DCF-DA at 530 nm is gradually enhanced with increasing the irradiation time (Fig. 9a and S19 of ESI†). Such change, however, was not observed in TPE-IQ-2O or H_2_DCF-DA alone. The efficiency of ROS generation of TPE-IQ-2O inside the cells was then investigated. As shown in Fig. 9b, the fluorescent images become brighter along with the irradiation time, indicating that a higher dose of ROS is generated.

**Fig. 10:**
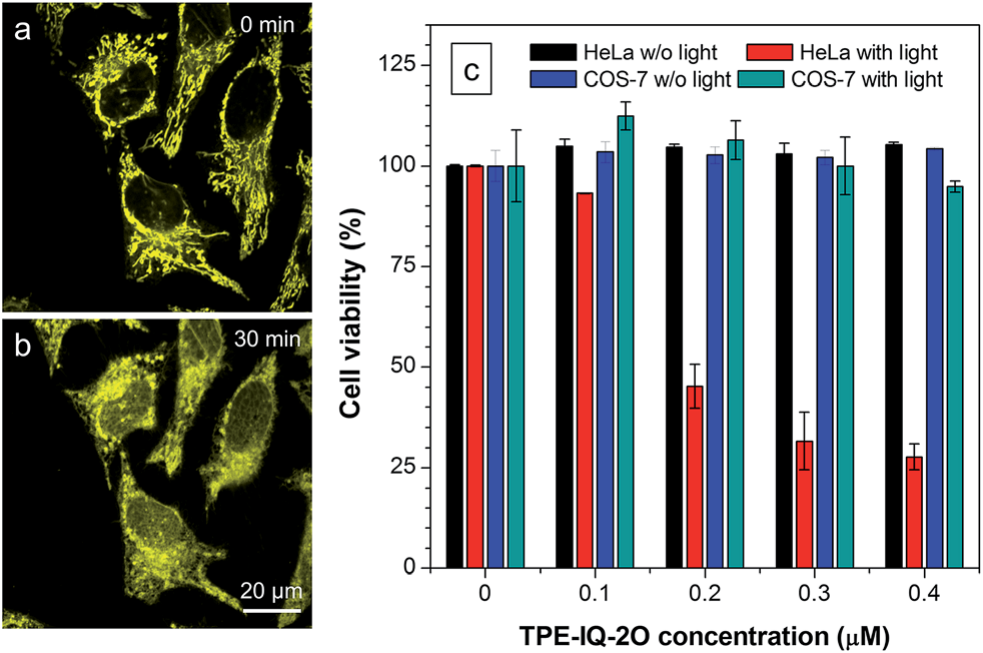
TPE-IQ-2O selectively kills cancer cells through PDT. (a and b) Change in mitochondrial morphology before and after white light irradiation. (c) Cell viability of HeLa cells and COS-7 cells stained with different concentrations of TPE-IQ-2O in the absence or presence of white light irradiation.

Excessive amount of ROS is detrimental to cells.^33^ As demonstrated in Fig. 10a and b, the mitochondria of dye-stained HeLa cells turn into irregular puncta after white light illumination, indicating that the ROS generated from TPE-IQ-2O can cause damage to the mitochondria and lead to cell death eventually. A LDH assay was thus applied to check the cell viability (Fig. 10c). In the absence of TPE-IQ-2O, the white light illumination brings almost no change in the cell viability. The cell viability of HeLa cells stained with TPE-IQ-2O, however, decreases upon white light irradiation. The higher the concentration of TPE-IQ-2O used, the lower is the cell viability. Noteworthy, no obvious change in the cell viability was observed when the dye-stained cells were kept in the dark or far away from light irradiation, irrespective of the dye concentration used for staining. This indicates that the simultaneous combination of TPE-IQ-2O and light irradiation will bring therapeutic effect to the cancer cells.

To demonstrate the ability of TPE-IQ-2O for selectively killing cancer cells, we did the same experiment by using COS-7 cells. As expected, almost no toxic effect on COS-7 cells was found even when the cells were incubated in concentrated TPE-IQ-2O solution with light irradiation. This promising result suggests a good theranostic system that can selectively stain the mitochondria in cancer cells and induce their death through photodynamic therapy.

### Potential applications of theranostic AIEgens

Theranostics, a combination of diagnosis and therapy, is one of the most popular research topics in medical science. Thanks to their strong solid-state emission and superior photostability, AIEgens have demonstrated good performance in organelle imaging with high specificity and sensitivity. By integration of the PDT property, an AIE-based theranostic system will be developed. TPE-IQ-2O, could be a promising theranostic agent with AIE characteristics. It not only can preferentially stain cancer cells over normal cells, but also induce ROS to kill cancer cells upon white light irradiation without damaging the normal cells.

Surgical operation is a common approach to remove tumor cells. During the surgery, the experience of the surgeon remains the dominant element to decide which part of the tissues should be resected even with the help of preoperative imaging techniques.^34^ Hence, the complete removal of cancer cells remains a challenge for surgical operation due to the irregular margins of tumors.^35^ Due to its property of selective illumination of cancer cells, TPE-IQ-2O can be used as a guiding-agent in surgery. It can provide much clearer margins between cancer and normal tissues than traditional imaging methods owning to its high brightness and fluorescence contrast. In addition, the residual cancer cells can be specifically lysed upon white light irradiation, preventing the reoccurrence of the disease.

Apart from imaging-guided therapy, TPE-IQ-2O may also find application in circulating tumor cell (CTC) detection. CTC from the peripheral blood of patients are used as a cancer biomarker in more than 270 clinical trials.^36^,^37^ CTCs occur at a very low concentration, with only 1–10 cells per mL of blood in most cancer patients. Thus, it is challenging to separate and recognize these rare cells specifically from millions of healthy blood cells. The commonly used techniques for CTC isolation rely on antibodies against epithelial cell-adhesion molecule.^38^ However, this strategy is expensive and complicated. Due to the fact that it can selectively distinguish a small number of cancer cells from a large population of normal cells, TPE-IQ-2O is therefore a promising tool for the search of rare CTCs in the peripheral blood samples.

## Conclusions

In summary, we have developed a theranostic system with AIE characteristics that can selective stain and kill cancer cells. TPE-IQ-2O is an example as demonstrated in this work. It is a mitochondrion-specific probe with greenish-yellow emission; meanwhile, it can selectively target the cancer cells but not normal ones. Mechanistically, the specific localization of TPE-IQ-2O on mitochondria depends on the MMP of cancer cells. Additionally, TPE-IQ-2O can generate ROS efficiently within cancer cells to induce cell death upon white light irradiation. The combination of cancer cell recognition and therapy make TPE-IQ-2O a promising theranostic agent. More importantly, the high fluorescence contrast between the cancer and the normal cells allows their differentiation by TPE-IQ-2O, making the dye useful in imaging-guide surgery. Currently, we are working on developing more theranostic systems based on AIEgens with longer absorption and emission wavelengths and higher PDT efficiency. Details will be reported in the due course.

## Experimental

### Materials and instruments

All chemicals and reagents were used as received useless otherwise specified. 2′,7′-Dichlorodihydrofluorescein diacetate (H_2_DCF-DA), oligomycin A and carbonyl cyanide *m*-chlorophenyl hydrazine (CCCP) were purchased from Sigma-Aldrich. Minimum essential medium (MEM), Dulbecco's modified eagle medium (DMEM), Roswell Park Memorial Institute (RPMI)-1640 medium, phosphate buffered saline (PBS), fetal bovine serum (FBS), penicillin and streptomycin, MitoTracker® Red, LysoTracker® Red DND-99, 4′,6-diamidino-2-phenylindole (DAPI) were purchased from Invitrogen. The Pierce™ LDH Cytotoxicity Assay Kit was a commercial product of Thermo Scientific. Milli-Q water was supplied by Milli-Q Plus System (Millipore Corporation, United States). Tetrahydrofuran (THF) was distilled from sodium benzophenone ketyl under nitrogen immediately prior to use. Silver tetrafluoroborate (AgBF_4_) was purchased from J&K Scientific. Diphenylacetylene, propylamine, dimethylsulfoxide (DMSO), copper(ii) acetate [Cu(OAc)_2_] and other chemicals as well as solvents were all purchased from Aldrich.

^1^H and ^13^C NMR spectra were measured on a Bruker ARX 400 NMR spectrometer using chloroform-d as the solvent and tetramethylsilane (TMS; *δ* = 0) as the internal reference. High-resolution mass spectra (HRMS) were recorded on a Finnigan MAT TSQ 7000 Mass Spectrometer System operating in a MALDI-TOF mode. Absorption spectra were taken on a Milton Ray Spectronic 3000 array spectrophotometer. Steady-state photoluminescence (PL) spectra were recorded on a Perkin-Elmer spectrofluorometer LS 55. Fluorescent images were collected on Olympus BX 41 fluorescence microscope. Confocal images were collected on a Zeiss laser scanning confocal microscope (LSM7 DUO) and analyzed using ZEN 2009 software (Carl Zeiss). The fluorescence intensity from flow cytometry was obtained from a Becton Dickinson FACSAria IIIu.

### Synthesis and characterization of TPE-IQ-2O

Compound 2 was synthesized according to the reported literature.^39^^1^H NMR (400 MHz, CDCl_3_), *δ* (TMS, ppm): 9.90 (s, 1H), 7.61 (d, 2H), 7.18–7.12 (m, 5H), 7.00 (d, 2H), 6.93 (d, 4H), 6.64 (d, 4H), 3.75 (d, 6H). ^13^C NMR (100 MHz, CDCl_3_), *δ* (TMS, ppm): 191.8, 158.5, 158.4, 151.2, 143.5, 142.3, 137.9, 135.6, 135.5, 133.9, 132.6, 132.5, 131.9, 131.3, 129.1, 127.9, 126.5, 113.2, 113.0, 55.0. MALDI-TOF: calcd for C_29_H_24_O_3_: *m*/*z* 420.1725, found: 420.1730.

Into a reaction tube under N_2_ atmosphere were dissolved [RhCp*Cl_2_]_2_ (2.0 mol%),^40^,^41^ Cu(OAc)_2_ (0.30 mmol), AgBF_4_(0.30 mmol), diphenylacetylene (0.30 mmol) and 2 (0.36 mmol) in a solution of propylamine (0.45 mmol) and *t*-amyl alcohol (2.5 mL). The mixture was heated to 110 °C and stirred for 3 h. Afterwards, the reaction mixture was diluted with dichloromethane (10 mL) at room temperature and filtered through a Celite pad. The pad was then washed with a solution of DCM and methanol (3 : 2, v/v). The filtrate was condensed under reduced pressure and loaded onto alumina for column chromatography using a mixture of DCM/methanol as the eluent. TPE-IQ-2O was obtained as a yellow solid. Yield: 79.1%. ^1^H NMR (400 MHz, CDCl_3_), *δ* (TMS, ppm): 11.35 (s, 1H), 8.66 (d, 1H), 7.53 (d, 2H), 7.31–6.58 (m, 21H), 4.73 (m, 4H), 3.72 (s, 3H), 3.81 (s, 3H), 1.90 (d, 2H), 0.87 (m, 3H). ^13^C NMR (100 MHz, CDCl_3_), *δ* (TMS, ppm): 159.1, 154.6, 151.1, 145.5, 143.4, 142.7, 138.2, 137.4, 137.0, 135.1, 135.0, 134.6, 133.1, 133.0, 132.9, 131.6, 131.5, 131.1, 130.5, 130.2, 128.9, 128.5, 128.4, 127.3, 126.0, 114.0, 113.3, 60.3, 55.6, 55.3, 25.6, 11.0. MALDI-TOF: calcd for C_46_H_40_NO_2_^+^: *m*/*z* 638.3059, found: 638.3088; calcd for BF_4_: *m*/*z* 87.0035, found: 86.9980.

### Cell culture and cell imaging

HeLa cells and PC-9 cells were cultured in MEM and RPMI-1640 media, respectively. DMEM was used to culture MDA-MB-231, MCF-7, A549, HepG2, HCC827, HEK-293, LX2, COS-7 and MDCK-II cells. For co-culture experiments, the cells were cultured in DMEM. All the cells were cultured in media supplemented with 10% heat-inactivated FBS, 100 units per mL penicillin and 100 μg mL^–1^ streptomycin, in a humidity incubator with 5% CO_2_ at 37 °C. Before the experiment, the cells were pre-cultured until confluence was reached.

All the cells were grown overnight on a 35 mm Petri dish with a cover slip or a plasma-treated 25 mm round cover slip mounted to the bottom of a 35 mm Petri dish with an observation window. The live cells were incubated with 200 nM of TPE-IQ-2O (4 μL of a 0.1 mM stock solution of TPE-IQ-2Q in DMSO was diluted to 2 mL with culture media) for 10 or 20 min, and then were imaged using fluorescence microscopy. For imaging dead cells, the live cells were first treated with 12 μM of H_2_O_2_ and then incubated with TPE-IQ-2O (200 nM) for 20 min. For co-staining experiments, the cells were co-stained with TPE-IQ-2O (200 nM) and commercial biomarkers (MitoTracker Red (50 nM)/LysoTracker Red DND-99 (50 nM)/DAPI (500 nM)) for 20 min. The cells were then imaged using a fluorescence microscope (BX41 microscope) using a combination of different excitation and emission filters for each dye: for TPE-IQ-2O, excitation filter = 400–440 nm, dichroic mirror = 455 nm, and emission filter = 465 nm long pass; for MitoTracker Red and LysoTracker Red DND-99, excitation filter = 540–580 nm, dichroic mirror = 600 nm, and emission filter = 610 nm long pass; for DAPI, excitation filter = 330–385 nm, dichroic mirror = 400 nm, and emission filter = 420 nm long pass. For imaging using a laser-scanning confocal microscope, the fluorescence signal of the cells stained with JC-1 was collected using a 488 nm with 561 nm laser as excitation light. The spectral collection region was 515–545 nm and 575–625 nm, respectively. For the ROS generation experiment, the cells were first incubated in a mixture of TPE-IQ-2O (1 μM) and H_2_DCF-DA (1 μM). All the images were then collected at different times of white light irradiation using a 488 nm laser as excitation light. The spectral collection region was 500–550 nm. For observing the morphology change in mitochondrion, the cell images were continuously collected using 405 nm laser as excitation light. The spectral collection region was 420–600 nm.

### Optimization of staining conditions

The optimal staining conditions to achieve maximum emission contrast between HeLa cells and COS-7 cells were investigated. Cancerous HeLa cells and normal COS-7 cells were used as models and incubated with different concentrations of TPE-IQ-2O (50/100/200/500/750/1000 nM) for 20 min. As shown in Fig. S7 and S8,† an obvious fluorescence difference between the two types was observed in all experiments, in which 200 nM and 500 nM displayed the highest emission contrast. Further experiments then focused on concentrations with 200 nM and 500 nM for different incubation times (1/5/10/20/30/45/60 min). Compared to the later concentration, the first one seem to be more promising because the fluorescence recorded from the dye stained cells kept at a stable level when at a longer incubation time (see ESI, Fig. S9–S12†). Thus, 200 nM was chosen as the working concentration. In addition, the staining time was fixed at 20 min as this value was long enough to clearly differentiate cancer cells from normal cell.

### Cytotoxicity evaluation by LDH assay

The optimal concentration of HeLa or COS-7 cells was determined to be 5000 per well in a 96-well plate, following the standard method given by Thermo Scientific. To decrease the spontaneous LDH activity, culture media containing 2% FBS were used. After 24 h cell culture, different concentrations of TPE-IQ-2O were added into the 96-well plate. After 1 h incubation, two plates containing HeLa and COS-7 cells were exposed to white light (5 W) for 90 min, and another two plates with cells were kept in dark as control. After another 24 h cell culture, 10 μL of 10× lysis buffer bromide was added to untreated cells at the maximum LDH activity. After incubation at 37 °C, 5% CO_2_ for 45 min, 50 μL of each sample medium was transferred to a new 96-well plate followed by addition of 50 μL of LDH reaction mix. A positive control was included. After 30 min incubation, protected from light, 50 μL of stop solution was added to each well. The absorbance was read with a plate reader, from which the cytotoxicity of fluorophores could be determined.

### Adjustment of mitochondrial membrane potential (MMP)

HeLa cells were pre-treated or post-treated with 20 μM CCCP or 10 μg mL^–1^ oligomycin for 30 min before staining with 5 μM TPE-IQ-2O for 30 min. All the data were obtained from flow cytometry in the same collection region (*λ*_ex_: 488 nm, *λ*_em_: 515–545 nm).

### MMP quantification by JC-1

For measuring MMP of different cells using JC-1, all fluorescent images were taken on a confocal microscope using green (*λ*_ex_: 488 nm, *λ*_em_: 515–545 nm) and red channels (*λ*_ex_: 561 nm, *λ*_em_: 575–590 nm). Other intensity data were measured by MATLAB R 2010b.

## Supplementary Material

Supplementary information
