# Supplementary material for: AIE-active theranostic system: selective staining and killing of cancer cells
Source: Chem Sci. 2016 Dec 13;8(3):1822–30. doi: 10.1039/c6sc04947h (PMC6092713; doi:10.1039/c6sc04947h)
Supplement: Supplementary file 1 [file SC-008-C6SC04947H-s001.pdf]

Electronic Supplementary Material (ESI) for Chemical Science.
This journal is © The Royal Society of Chemistry 2016

Electronic Supplementary Information

AIE-active theranostic system: selective staining and killing of cancer Cells

*Chen Gui, Engui Zhao, Ryan T. K. Kwok, Anakin C. S. Leung, Jacky W. Y. Lam, Meijuan Jiang, Haiqin Deng, Yuanjing Cai, Weijie Zhang, Huifang Su and Ben Zhong Tang**

1. Chemical structure and cell imaging of TPE-IQ

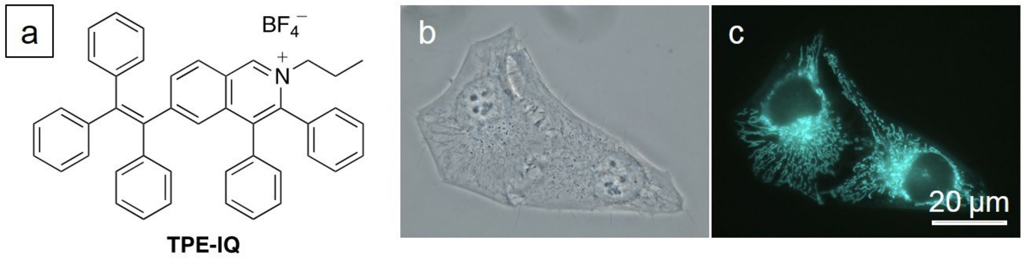

Fig. S1 (a) Chemical structure of TPE-IQ. (b) Bright-field and (c) fluorescent images of HeLa cells incubated with 200 nM of TPE-IQ for 20 min.

2. Synthetic route and characterization of TPE-IQ-2O

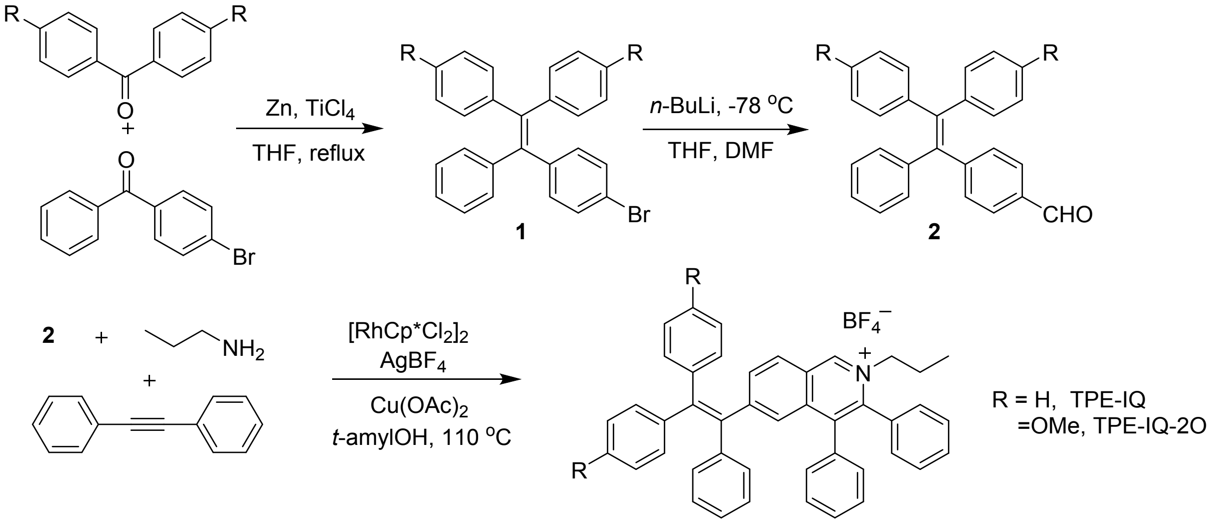

Fig. S2 Syntheses of TPE-IQ and TPE-IQ-2O.

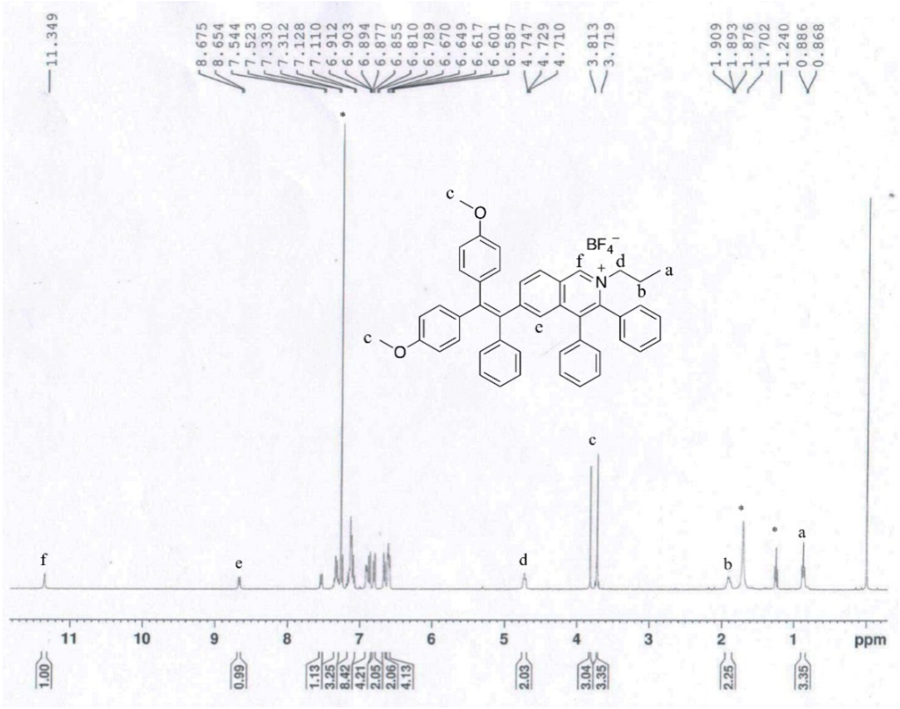

Fig. S3 ^{1}H NMR spectrum of TPE-IQ-2O in $CDCl_3$.

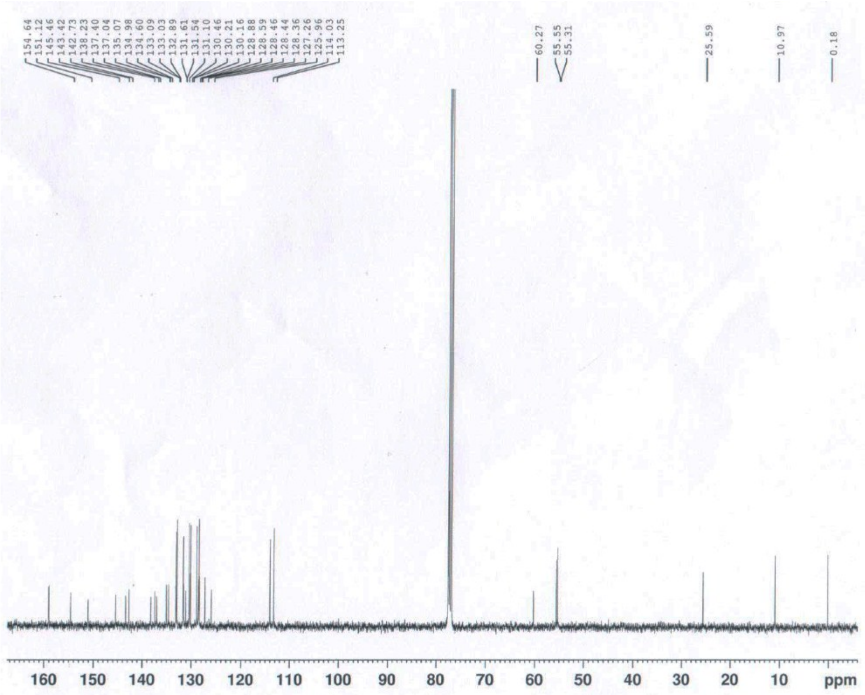

Fig. S4 ^{13}C NMR spectrum of TPE-IQ-2O in $CDCl_3$.

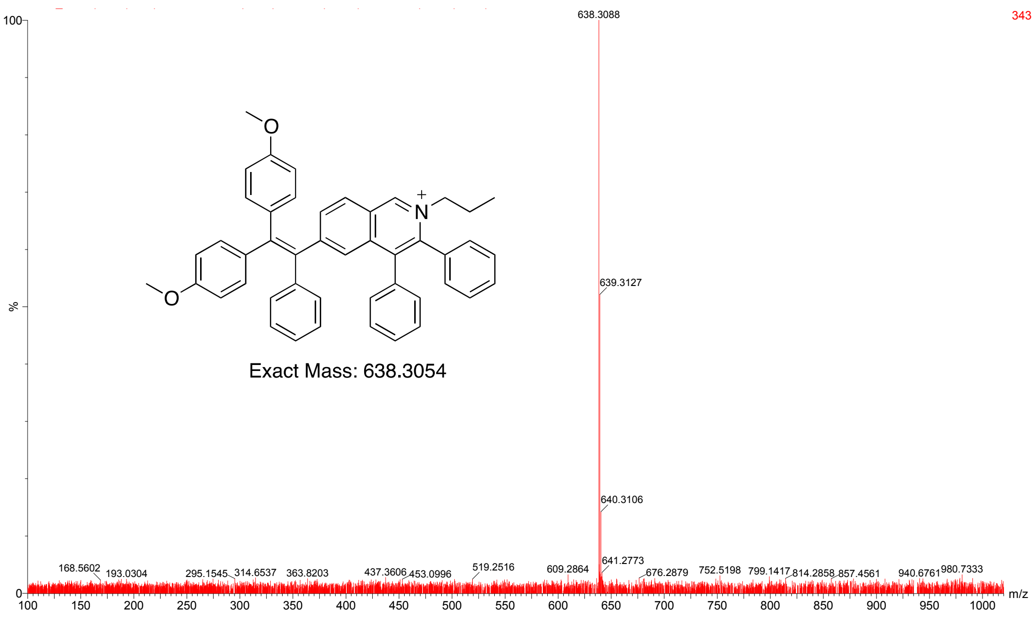

Fig. S5 High resolution mass spectrum of TPE-IQ-2O.

3. HeLa cells co-stained with LysoTracker Red/ DAPI and TPE-IQ-2O

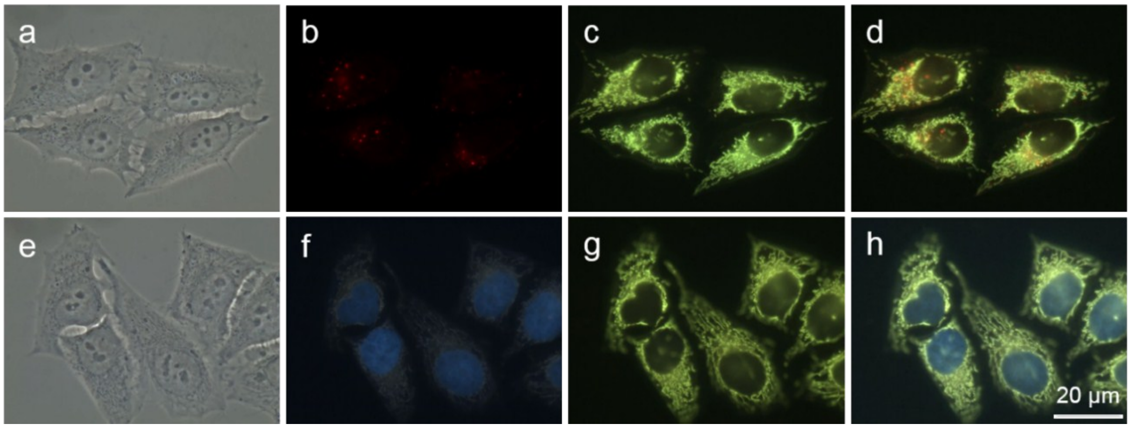

Fig. S6 (a) Bright-field and (b and c) fluorescent images of HeLa cells co-stained with (b) LysoTracker Red and (c) TPE-IQ-2O for 20 min. (d) The merged image of panel (b) and (c). (e) Bright-field and (f and g) fluorescent images of HeLa cells co-stained with (f) DAPI and (g) TPE-IQ-2O for 20 min. (h) The merged image of panel (f) and (g). Concentrations: TPE-IQ-2O (200 nM); LysoTracker Red (50 nM); DAPI (500 nM). Scale bar = 20 μm.

4. optimazation of staining conditions

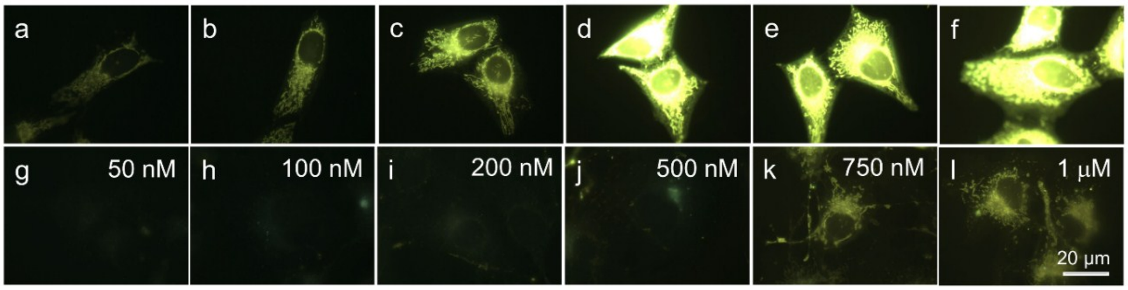

Fig. S7 Fluorescent images of HeLa cells and COS-7 incubated with different concentrations of TPE-IQ-2O. Fluorescent images of (a–f) HeLa cells and (g–l) COS-7 cells stained with (a and g) 50 nM, (b and h) 100 nM, (c and i) 200 nM, (d and j) 500 nM, (e and k) 750 nM and (f and l) 1 μM of TPE-IQ-2O for 20 min. Scale bar = 20 μm.

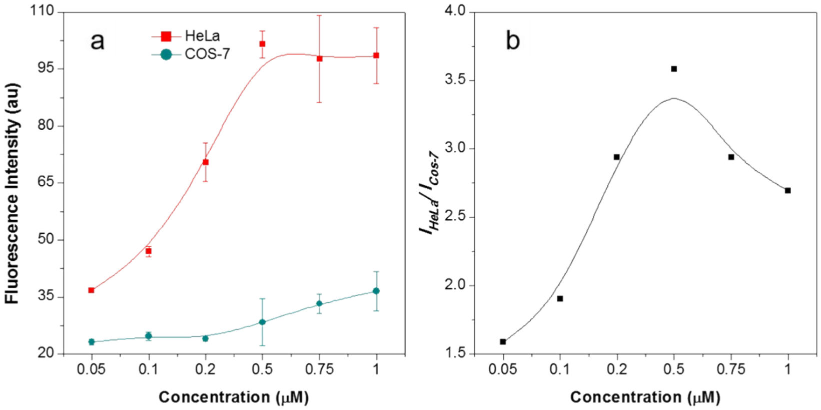

Fig. S8 Fluorescence difference between HeLa cells and COS-7 cells incubated with different concentrations of TPE-IQ-2O. (a) Fluorescence intensity from HeLa cells (red) and COS-7 cells (green) incubated with different concentrations of TPE-IQ-2O for 20 min. (b) Plot of relative fluorescent intensity (I_{HeLa}/I_{COS-7}) verse concentration of TPE-IQ-2O.

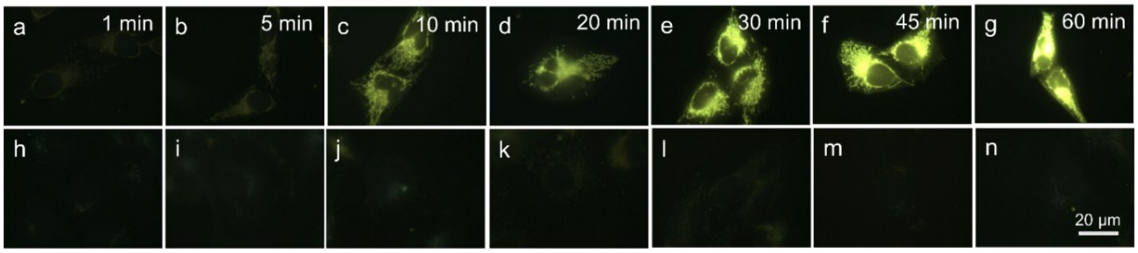

Fig. S9 Fluorescent images of HeLa cells incubated with 200 nM TPE-IQ-2O for different times. Fluorescent images of (a–g) HeLa cells and (h–n) COS-7 cells stained with 200 nM of TPE-IQ-2O for different times. Scale bar = 20 μm.

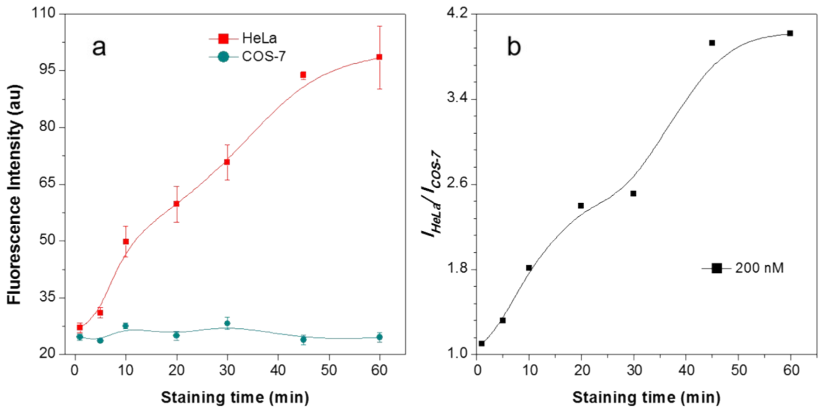

Fig. S10 Fluorescence difference between cancerous HeLa cells and normal COS-7 cells incubated with 200 nM TPE-IQ-2O for different times. (a) Fluorescence intensity from HeLa cells (red) and COS-7 cells (green) stained with 200 nM of TPE-IQ-2O for different times. (b) Plot of relative fluorescent intensity (I_{HeLa}/ I_{COS-7}) verse the staining time.

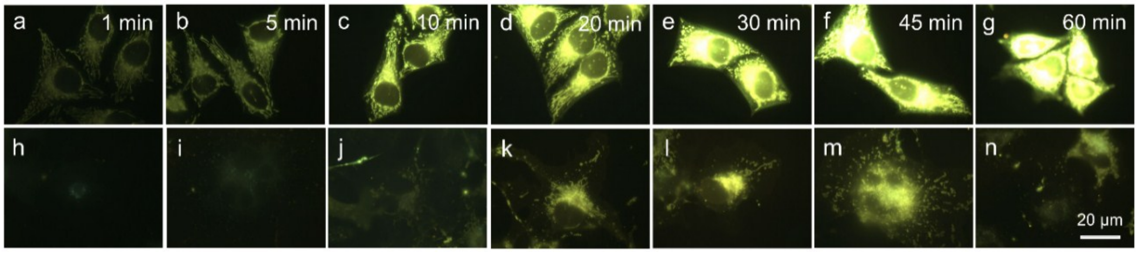

Fig. S11 Fluorescent images of HeLa cells incubated with 500 nM TPE-IQ-2O for different times. Fluorescent images of (a–g) HeLa cells and (h–n) COS-7 cells stained with 500 nM of TPE-IQ-2O for different times. Scale bar = 20 μm.

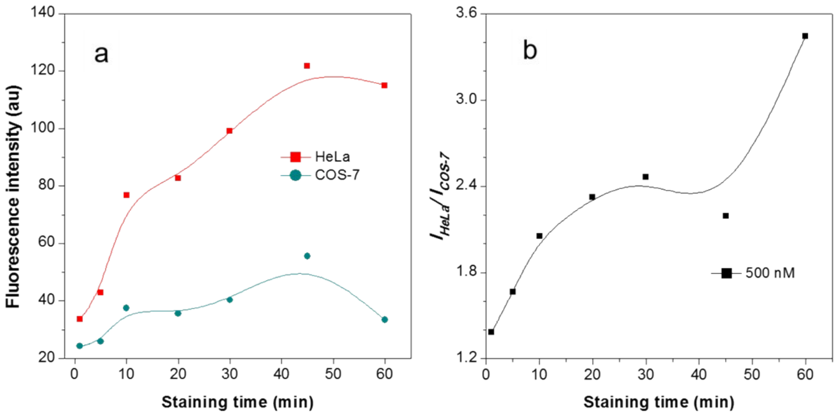

Fig. S12 Fluorescence difference between HeLa cells and COS-7 cells incubated with 500 nM TPE-IQ-2O for different times.(a) Fluorescence intensity from HeLa cells (red) and COS-7 cells (green) stained with 500 nM of TPE-IQ-2O for different times. (b) Plot of relative fluorescent intensity (I_{HeLa}/ I_{COS-7}) verse the staining time.

5. Fluorescent images of HCC827, HepG2 and LX-2 cells stained with TPE-IQ-2O

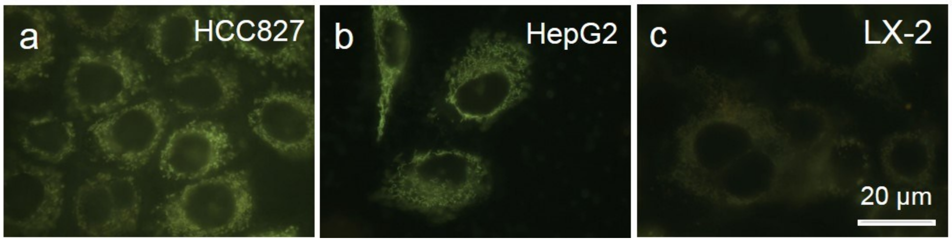

Fig. S13 Fluorescent images of different (a and b) cancerous cells and (c) normal cells stained with 200 nM TPE-IQ-2O for 20 min. Scale bar = 20 μm.

6. Images of HeLa cells co-cultured with COS-7 cells in different number ratios in the presence of TPE-IQ-2O

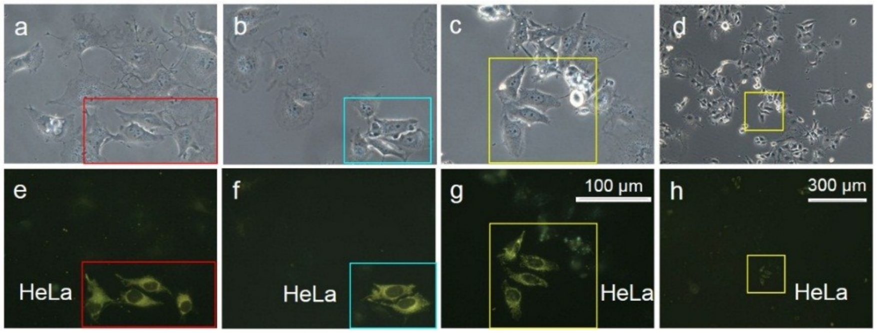

Fig. S14 (a–d) Bright-field and (e–h) fluorescent images of HeLa cells co-cultured with COS-7 cells in number ratios (HeLa/COS-7) of (a and e) 1:5, (b and f) 1:50, (c, d, g and h) 1:100 in the presence of TPE-IQ-2O for 20 min. (d and h) Contractible images of (c and g). Rectangle represents the location of HeLa cells. Scale bar = 100 μm.

7. Fluorescence intensity of HeLa cells with different MMP

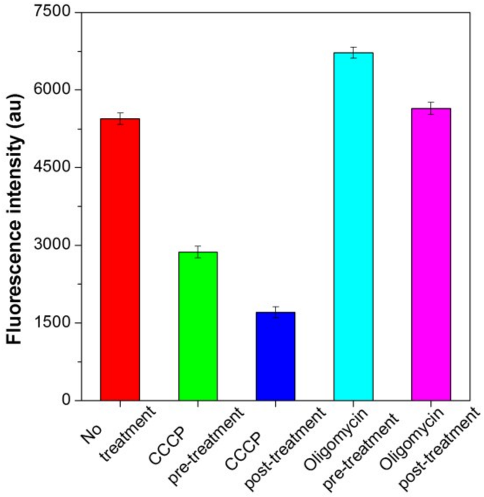

Fig. S15 Fluorescent intensity of HeLa cells stained with TPE-IQ-2O of different treatment.

8. Fluorescent images of live and dead HeLa cells stained with TPE-IQ-2O

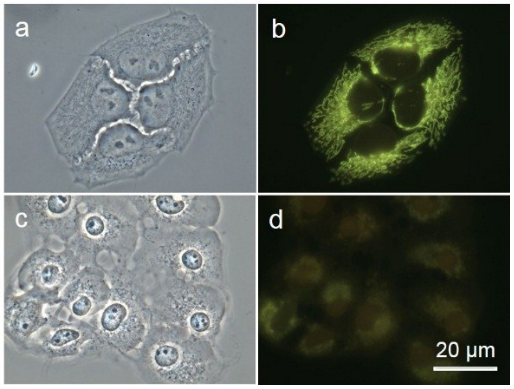

Fig. S16 (a and c) Bright-field and (b and d) fluorescent images of HeLa cells stained with TPE-IQ-2O (200 nM) for 20 min (a and b) without and (c and d) with pretreatment of H_2O_2. $[H_2O_2]$ = 2 mM.

9. Comparision of MMP in different cells

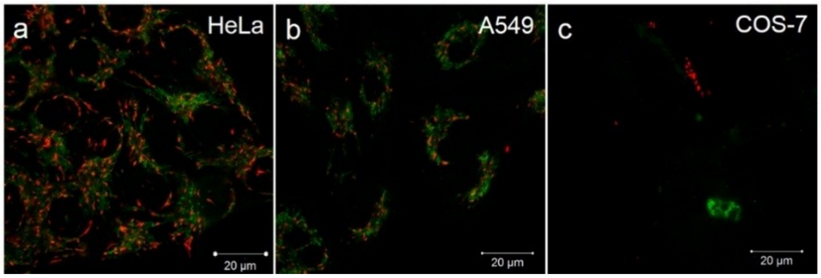

Fig. S17 Confocal images of (a) HeLa cells, (b) A549 cells and (c) COS-7 cells incubated with JC-1 (2 μM) for 30 min. λ_{ex}: 488 nm.

Table S1. Summary of fluorescence intensity of different cells stained with JC-1 (2 μM) at excitation wavelength of 405 nm (green channel) and 488 nm (red channel)

Cell Type	Red channel	Green channel	Red/Green
HeLa	25,079	43,443	1.7322
A549	14,824	22,127	1.4926
COS-7	36,713	12,231	0.3332

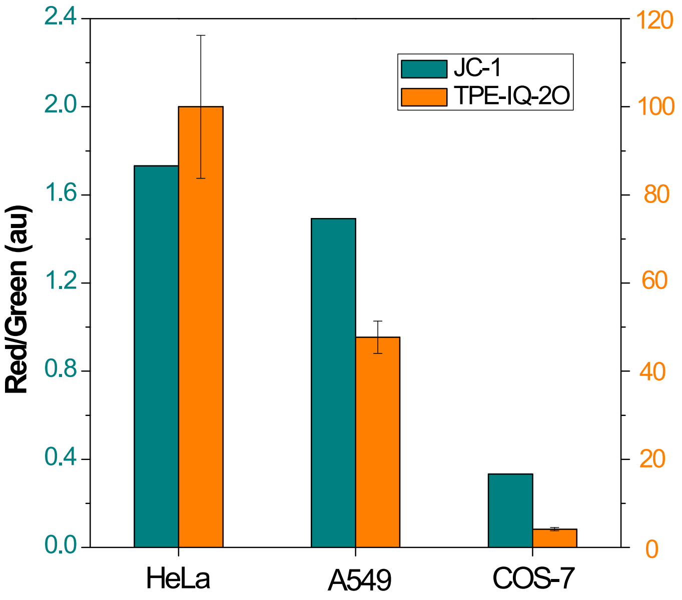

Fig. S18 Comparison between fluorescent intensity of different cells incubated with JC-1 and TPE-IQ-2O. (Green) Relative fluorescence intensity (red/green channel) of different cells stained with JC-1 (2 μM) at excitation wavelength of 405 nm (green channel) and 488 nm (red channel) and (Orange) fluorescence intensity of different cells stained with TPE-IQ-2O (200 nM) for 20 min.

10. TPE-IQ-2O works as a photosensitizer to generate ROS

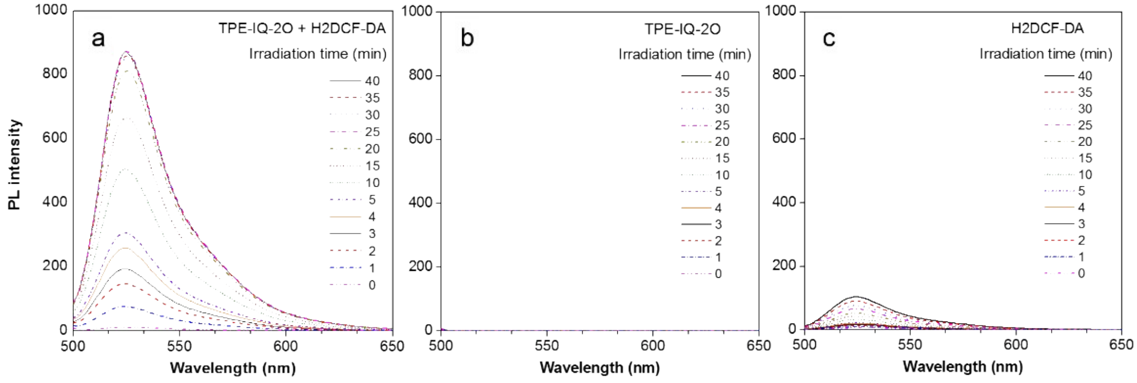

Fig. S19 (a–c) PL spectra of (a) a mixture of TPE-IQ-2O and H2DCF-DA, (b) TPE-IQ-2O solution and (c) H2DCF-DA solution obtained under light irradiation for different times. Concentration: 10 μM (TPE-IQ-2O) and 5 μM (H2DCF-DA); λ_{ex}: 488 nm.
